# Order effect of repair processes to clustered DNA damage

**DOI:** 10.1093/jrr/rrt152

**Published:** 2014-03

**Authors:** Iyo Shiraishi, Masao Suzuki, Naoya Shikazono, Kentaro Fujii, Akinari Yokoya

**Affiliations:** 1 Ibaraki University, Japan; 2 JAEA, Japan; 3 NIRS, Japan

**Keywords:** clustered DNA damage, base excision repair, high LET radiation, carbon ion beam

## Abstract

In a living cell, a multiply damaged site in DNA is thought to be processed by several different pathways simultaneously or sequentially. Under this situation, the cellular response to the lesion cluster might depend on the order of repair processes because the configuration of the lesions will be modified by the reaction of initial repair protein, affecting the DNA-binding or lesion-excision activities of latter repair proteins. For example, a cluster comprised an AP site or SSB and base lesions is formed after one of the lesions in a base lesion cluster is excised by a glycosylase. It has been predicted that the enzymatic activity of a protein binding to a lesion could be affected by a second lesion located nearby. In the present study, we investigate whether initial base excision repair process affects secondary process on the clustered DNA damage site produced by high linear energy transfer irradiation.

Plasmid DNA (pUC18) used as model DNA were irradiated in a solution (10 mM Tris and 1 mM EDTA) with C^6+^ ions (60 keV/µm) obtained from HIMAC (NIRS, Chiba, Japan) according to our previous method [ 1], or He^2+^ ions from TIARA (JAEA, Takasaki, Japan) in a fully hydrated film [ 2]. We used two base excision repair enzymes to examine the order effect of BER processes, Nth and Fpg, which convert pyrimidine and purine lesions to an SSB, respectively. After exposure, the sample was incubated with Nth (or Fpg) at 37°C for 30 min. Then, we added another enzyme Fpg (or Nth) and incubate for another 30 min. We also simultaneously treated the DNA sample with both enzymes for 1 h. After the incubation, the samples were analysed by agarose (1%) gel electrophoresis to determine the yield of conformational changes of the plasmid from closed circular form to open or linear form. These changes show productions of an enzymatically induced SSB (the former) or DSB (the latter).

A typical example of the dependence of the loss of closed circular form DNA on radiation dose is shown in Fig. 1a for carbon ion irradiation. The reciprocal of the slope of each graph shows yield of prompt SSBs [*n*(SSB)_prompt_] for the sample without any enzymatic treatment, or [*n*(SSB)_prompt_] + enzymatically induced SSBs [*n*(SSB)_Nth(or Fpg, or Nth+Fpg)_]. The SSB yield for the sample treated with Nth first and then Fpg is very slightly less than that of other treatments (<10%). The dose responses of the linear form DNA production are shown in Fig. 1b. There were no significant differences among the enzymatic treatments. These results indicate that the configuration change of the cluster by the first enzymatic treatment does not significantly influence the activity of secondary enzyme. The most of base lesion clusters produced in the track of carbon ions might be converted to strand breaks, some of which form double-strand breaks (DSBs), by glycosylase activity in living cells.

**Fig. 1.**
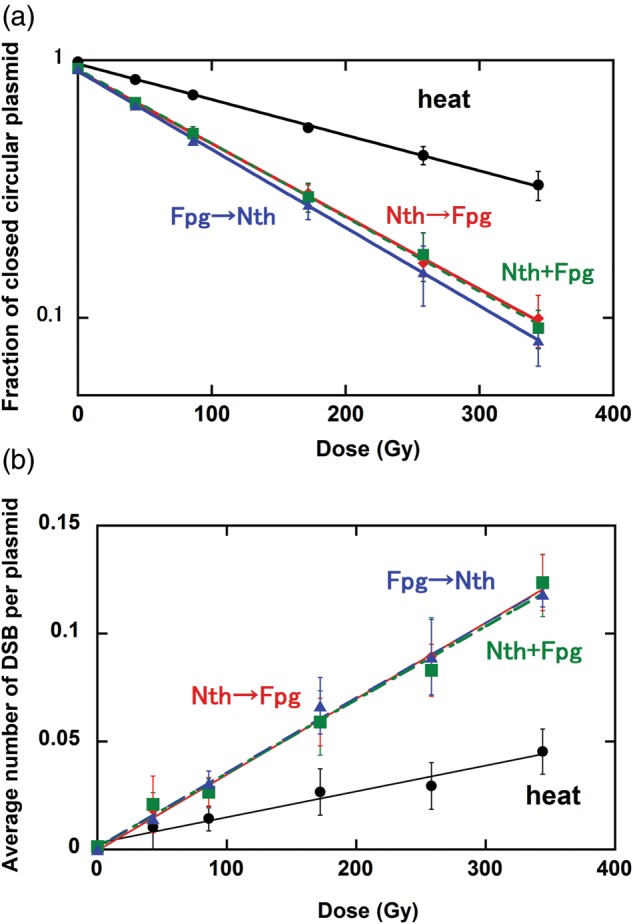
Dependence of (a) the loss of closed-circular DNA and (b) the number of linear form DNA DSB determined from the fraction of the linear form of DNA, on carbon ion dose. The arrows indicate the order of the enzymatic treatments.

Dependence of (a) the loss of closed-circular DNA and (b) the number of linear form DNA DSB determined from the fraction of the linear form of DNA, on carbon ion dose. The arrows indicate the order of the enzymatic treatments.
